# Exploring a Critical Threshold in Sleep Loss on Subsequent Evening Performance, Following Two Nights Partial Sleep Restriction: A Secondary Analysis is Implementing a Post‐lunch Nap Beneficial?

**DOI:** 10.1002/brb3.70741

**Published:** 2025-08-12

**Authors:** Ben Joseph Edwards, Ellis Brotherton, Adrian Markov, Theresa Toussaint, Magali Giacomoni, Chloe Gallagher, Samuel Andrew Pullinger

**Affiliations:** ^1^ Research Institute for Sport and Exercise Sciences Liverpool John Moores University Liverpool UK; ^2^ Philipps‐Universität Marburg Marburg Germany; ^3^ UFR STAPS, Laboratoire J‐AP2S Université De Toulon Toulon France; ^4^ Sport Science Department Inspire Institute of Sport Vidyanagar India

## Abstract

**Purpose:**

We investigated sleep‐restriction (SR, two nights) on evening (5:00 p.m.) weightlifting and effects following a 1‐h nap. Thirty strength‐trained males were allocated into two groups, either 4‐h [SR_4_] or 3‐h sleep [SR_3_], retiring at either 02:30 or 03:30 p.m. and waking at 06:30 p.m. for two nights), with 5:00 p.m. grip‐strength and bench‐press; and whether performance improves at 5:00 p.m. after a 1:00 p.m. nap (0 or 60‐min).

**Method:**

Both groups undertook a one‐repetition‐max (1RM) for bench press and were randomly allocated either condition (i) no (N_0_) then (ii) a 60‐min nap (N_60_). Intra‐aural temperature/profile‐of‐mood‐scores/alertness/tiredness/sleepiness values were measured at 08:00 a.m., 11:00 a.m., 2:00 p.m., and 5:00 p.m. following the two nights of SR. At 5:00 p.m., a warm‐up, right‐hand grip strength, followed by lifts (40, 60, and 80% of 1RM) for bench press with recovery occurred. A linear encoder, attached to the bar, measured peak‐power (PP), peak‐velocity (PV), displacement (D) and time‐to‐peak velocity (tPV). Grip strength was not affected by SR or nap (*p* > 0.005). PP and PV were lower and tPV longer in the SR_4_ versus SR_3_ group, reflecting a higher 1RM. Lower tiredness/fatigue/confusion/sleepiness and an increase in alertness in the SR_4_ group than SR_3,_ reflecting effects of greater sleep loss on tiredness/alertness/sleepiness/mood. Time‐of‐day effects were shown in temperature/subjective/mood‐responses (*p* < 0.05), with positive modifications in sleepiness/alertness/tiredness after the 1‐h nap at 1:00 p.m. in both the SR_3_ an SR_4_ groups.

**Finding:**

Improvements in PV after the 1‐h nap were only found in the SR_3_ condition (*p* = 0.029). In summary, a dose‐response of sleep loss on subjective values and mood was found (SR_3_ worse than SR_4_). But not grip‐strength or bench‐press –strength being resistant to sleep loss and a stronger cohort in the SR_3_ than the SR_4_ group.

**Conclusion:**

Implementing a nap (N_60_) improved alertness/vigor/happiness and some strength measures (↑PV) for bench press compared to N_0_, but this was only effective for the SR_3_ condition.

## Graphical abstract

We have investigated the effects that two levels of sleep restriction (either 3 or 4‐h sleep during two consecutive nights) have on subsequent evening (17:00 h) submaximal weightlifting session; and whether this performance improves following a 1‐h post‐lunch powernap. We found a dose‐response of sleep loss on subjective values and mood was found (SR3 worse than SR4). But not grip strength or Bench press – reflecting maximal strength measures being resistant to sleep loss and a stronger and larger cohort in the SR3 than SR4 group. The implementing a nap (N60) at 13:00 h improved measures of alertness, vigour, happiness and some strength measures (↑PV) for bench press at 17:00 h compared to N0, but this was only effective for the SR3 condition.

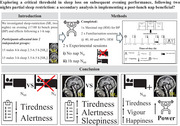

## 1 Introduction

Sleep is fundamental to animal life, as it has a restorative effect on many physiological systems, including immune and endocrine functions, to recover from the waking phase of the day, and it plays a role in learning and memory (Doherty et al., 2019). In humans, less sleep than needed leads to detrimental changes in cellular restitution, brain detoxification, growth, and repair (Watson et al., 2015). Similarly, more sleep than needed is also not recommended, as it would impair the active phase of the sleep‐wake cycle (Walsh et al., 2021). Sleep also plays a major role as a recovery strategy in athletes (Walsh et al., 2019; 2021). Partial sleep restriction (SR), that is a reduction in the habitual sleep within a 24‐h period is common in the western adult population, with around half taking less than recommended (7–9 h per night; Hirshkowitz et al., 2015; Craven et al., 2022). It is thought that athletes may exhibit poor sleep quality and duration due to demands of training/competition, as well as transmeridian travel challenging circadian rhythms and individual sleep phenotypes and environmental factors (de Mello et al. 2025). Resulting in sleep durations of ∼6.6 h per night, with poor sleep quality within this period (Sargent et al., 2014; Walsh et al., 2021; Craven et al., 2022; Simpson et al., 2017).

Circadian rhythm disturbances to the sleep‐wake cycle can be detrimental to physiological and psychological measures, resulting in reductions in cognitive function and some components of sport, with greater negative effects the more sleep loss (Reilly and Edwards, 2007; Souissi et al., 2008). The effects of sleep loss on cognition have been extensively investigated (Thun et al., 2015), and to a lesser extent so has sporting performance (Leeder et al., 2012; Walsh et al., 2021). A dose‐response to sleep loss has been proposed, where different amounts of sleep loss (compared to habitually taken per night) for a number of nights (7–14 days) as well as short‐term sleep restriction (24–48 h) have been shown to negatively affect mood, performance in psychomotor vigilance tests or cognitive performance (attention, working memory, and executive function), metabolic function, and psychological health (Van Dongen et al., 2004, 2003; Belenky et al., 2003; Banks et al., 2010; Lim & Dinges, 2010; Goel et al., 2009).

Belenky et al. (2003) examined the cumulative effects of chronic sleep restriction by assigning participants to one of four sleep duration conditions (3, 5, 7, or 9 h per night) for seven consecutive nights, followed by a three‐day recovery period. Results indicated that psychomotor vigilance performance (PVP) declined in the 5‐ and 7‐h sleep conditions, with performance deficits stabilizing after the second day. In contrast, participants restricted to 3 h of sleep per night exhibited progressive and unremitting impairments in PVP across the full 7‐day period. The authors concluded that sleep durations below 4 h per night induce significant decrements in sustained attention and neurobehavioral performance. Notably, the study did not include assessments of sport‐specific or physical performance outcomes (such as submaximal muscular or weightlifting performance), where greater exposure to sleep loss leads to a correspondingly proportionally larger impact on performance output (or recent reviews, see Silva et al., 2021; Walsh et al., 2021).

It seems prudent to employ interventions to combat sleep loss; however, the effectiveness of such interventions (such as a nap or nutritional supplements such as caffeine or zinc magnesium aspartate) is conflicting (Walsh et al., 2021 **;** Silva et al., 2021; Gallagher et al., 2024; Edwards et al., 2024a, 2024b). This is thought to be due to differences in research design that investigations employ, such as reductions compared to habitual levels of sleep and timing of restriction (late bedtime, early rising; Brotherton et al., 2019). The implementation of a “nap” is considered a safe and non‐invasive intervention to overcome partial sleep loss that can help increase total sleep time over the 24‐h period and restore performance in athletes (Silva et al., 2021; Walsh et al., 2021).

Approximately 43% of athletes are reported to engage in some form of napping; however, the optimal timing of naps remains challenging to determine due to the constraints imposed by training and competition schedules (Lastella et al., 2015; Romyn et al., 2018). A nap between of 30–60 min between 11:00 a.m. and 3:00 p.m. is usually recommended, as it matches with a timeframe during which alertness decreases and sleepiness and fatigue increase. This is also a convenient period in an athlete’ daily schedule (Lastella et al., 2021; Edwards et al., 2023). Although optimal ’nap durations are still unknown, a 60‐min nap has previously been shown to improve evening weightlifting performance (Brotherton et al., 2019).

The impact of sleep deprivation on aspects of muscular performance is influenced by the time‐of‐day and the number of hours the person has been awake when the measurement is performed. Specifically, the homeostatic component, or the length of time since waking, can negatively affect the body's natural circadian rhythms. Particularly in areas such as motivation and mood, which in turn can diminish muscular performance. This detrimental effect of sleep drive becomes even more pronounced when training or competition occurs in the evening and when partial sleep restriction extends over multiple days. Typically, two to three, rather than being limited to just a single 24‐h period. Weightlifting efforts (as used by athletes in training and competition), using force‐velocity linear encoders to measure muscle force output at different masses (low and high) have been reported to be sensitive to detect diurnal variation in bench press, leg press and back squat (Robertson et al. 2018; 2024) as well as partial sleep deprivation (Brotherton et al. 2019). Hence, intervention strategies such as a nap or nutritional supplement have been investigated by this protocol (Brotherton et al., 2019; Gallagher et al., 2023; 2024; Edwards et al., 2024a).

Therefore, the purpose of the present study was to (1) determine the physiological and psychological effects of two modalities of repeated partial sleep restriction (two independent groups, 3 versus 4‐h of sleep per night over two consecutive nights; SR_3_ vs. SR_4_) on evening muscle performance as well as changes in mood state, intra‐aural temperature, tiredness, sleepiness, and alertness subjective values; and (2) investigate the effectiveness of a 60‐min post‐lunch nap (N_60_) in improving evening physiological and subjective psychological measures. It was hypothesized that the partially sleep‐restricted 3‐h condition (SR_3_) would have negative consequences to subjective measures of mood, tiredness, sleepiness, and alertness as well as decrease strength performance measures compared to the 4‐h sleep condition (SR_4_). We further hypothesized that the 60‐min nap opportunity, following two nights of consecutive sleep restriction, would lead to greater submaximal strength performance at 5: 00 p.m. compared to no nap (N_0_).

## 2 Methods

### 2.1 Participants

This was a secondary analysis of data from two studies investigating “is implementing a post‐lunch nap beneficial on evening performance,” following two nights of partial sleep restriction of 3 h (Brotherton et al., 2019) or 4 h (Gallagher et al., 2023). Thirty strength‐trained males, as identified by sex and gender (mean ± SD: 22.2 ± 2.2 years; body mass: 84.2 ± 13.1 kg; height: 179.7 ± 7.2 cm; normal daily retiring and rising times 23:20 ± 12:30 a.m. and 07:33 ± 12:51 a.m.; total opportunity to sleep ∼8.1 decimal h; Table 1), volunteered for this study and undertook either sleep restriction of 3 h (SR_3_, *n* = 15) or 4 h (SR_4,_ *n* = 15). Sample size was determined using power calculation software (G*Power, version 3.1.9.6), based upon a large effect size of 0.8 for AP with a power of 0.80 and an *α* = 0.05, which determined a sample of 12 participants was required. Similar effect sizes of 0.90 for AP have been reported by Brotherton et al. (2019), who employed similar techniques and study design. Fifteen participants were recruited to account for dropout in each group. In line with our inclusion criteria, participants were injury‐free with no diagnosed sleep disorders (reported as not having a sleep disorder) and had not completed shiftwork or travelled outside the local time‐zone in the past month—this information was obtained by a questionnaire. As part of the selection criteria, 7‐day sleep diary data explicitly reporting the average sleep/wake timing data on bedtime, wake time, and total sleep opportunity time for both SR groups was recorded the week before the experimental session was undertaken. The participants were asked if this information represented their normal sleep/wake habits otherwise to complete the diary when they were in a normal routine. In addition to reporting the sleep/wake timing data, the participants demographic characteristics including age was recorded. As well as interindividual factors, such as chronotype (a person's natural predisposition to be awake or asleep at certain times) or ability to shrug off fatigue (flexibility/rigidity of sleep habits), which can influence effects following sleep loss (Durmer and Dinges 2005) and a 5‐day dietary intake questionnaire for both SR groups. Full information of each group is presented in Table 1. A verbal explanation of the experimental procedure was provided; this included the aims of the study, the possible risks associated with participation, and the experimental procedures to be utilized. All participants had to express no preference to training regarding time‐of‐day. All participants gave their written informed consent. The experimental procedures were approved by the Human Ethics Committee at Liverpool John Moores University. The study was conducted in accordance with the ethical standards of the journal and complied with the Declaration of Helsinki.

**TABLE 1:** Mean ± SD values for SR3 and SR4 groups for age, height, mass, chronotype, flexibility/rigidity, languidity/vigorous questionnaire scores. As well as Habitual retiring and waking times and total opportunity to sleep (h) and 1 RM for bench press. NB: This information was taken before the main experiment as part of screening or familiarization.

	Age (yrs)	Height (m)	Mass (Kg)	Chronotype score	Flex./Regid.	Lang./Vig.	Habitual Retiring time (decimal h)	Habitual Waking time (decimal h)	Total sleep (decimal h)	1 RM Bench press (kg)
**SR3 group**	22.7±2.5	1.82±0.89	89.0±13.8	31.8±4.5	47.4±4.2	44.5±6.3	23.3±0.3	7.3±0.6	8.1±0.5	98.0±24.8
**SR4 group**	21.6±1.6	1.77±0.59	79.4±10.8	32.9±4.0	45.5±3.7	44.1±4.9	23.2±0.7	7.9±1.0	8.2±0.6	80.7±25.0

### 2.2 Research Design

**FIGURE 1:**
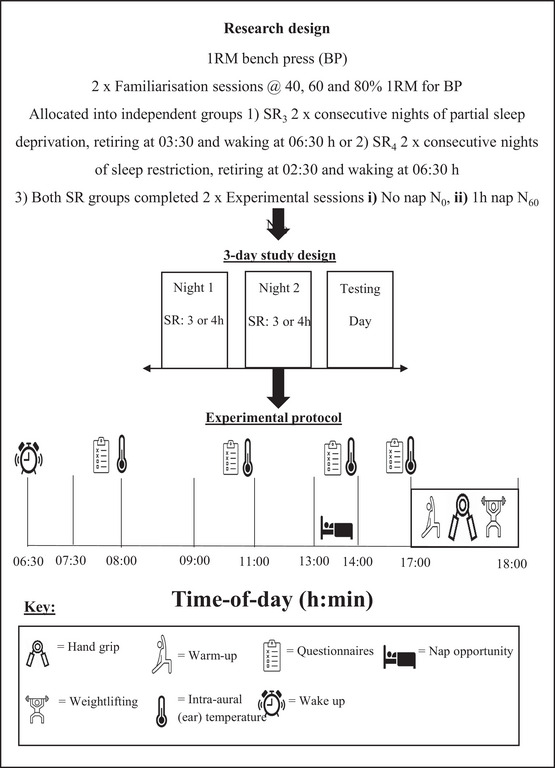
Schematic of experimental protocol. Participants followed the same procedures for each condition, with the addition of no nap (N_0_) or a 60‐min nap (N_60_) at 1:00 p.m. for the independent groups SR_3_ and SR_4_. At 5:00 p.m. participants entered the laboratory and undertook the performance measures.

Each participant attended the laboratory on five occasions (dry temperature of 19°C, 35–45% humidity, and a barometric pressure of 750–760 mmHg, respectively). All participants completed a habitual food and sleep diary two weeks prior to the first experimental condition to ensure all participants were maintaining healthy sleep habits prior to testing. The food diary was used to work out a normal calorific daily intake for that individual, and this was used to create a plan for foods that was replicated for the 2 days associated with sleep loss nights and the day of testing. Thereafter each participant completed (i) a bench press one repetition maximum (1RM), defined as the maximal weight that can be lifted once while maintaining correct technique (Kraemer et al., 1995) and (ii) two familiarization sessions. A 7‐day period separated the 1RM session and both familiarization sessions to allow adequate recovery. Each familiarization consisted of a standardized active warm‐up and three completed repetition lifts at 40, 60, and 80% 1RM for bench press (Figure 1). The first familiarization session involved collection of each participant's age, body mass, and height, followed by assessment of chronotype, etc. Following completion of the final familiarization, there was another 7‐day period before commencing the first experimental condition.

The two experimental conditions involved two nights of sleep taken at the participants home before entry into the laboratory on the third day (Figure 1). Sleep restriction consisted of retiring to sleep for two nights at 02:30 a.m. or 03:30 a.m. (depending on sleep restriction group SR_4_ or SR_3_) and rising at 06:30 a.m. and followed no nap (N_0_) or a 60‐min nap (N_60_) at 1 p.m. until 2:00 p.m. In the hour before retiring to sleep, participants were asked to refrain from watching television and/or usage of their mobile devices. Participants for both independent sleep restriction groups (SR_3_ and SR_4_) were required to complete the two nap conditions (N) in a counterbalanced order of administration to minimize any potential learning effects (Edwards et al., 2024c). When completing the N_60_ experimental conditions, participants were required to sleep/rest on a bed provided in a dark, quiet room in the university sleep laboratory and were not permitted to get up from the bed until the end of the 1‐h session (under the supervision of a researcher). Following the nap condition, participants were asked if they had “Cnapped” following this duration, with some participants stating, “They managed to sleep,” and others, “They rested their eyes.” At 1:00 p.m. in the N_0_ condition, participants were allowed to undertake free living conditions and were instructed not to nap or exercise. Researchers checked in with participants via direct messages to ensure sleep compliance the day of the experiment and the two nights leading to the laboratory visit. As well as that they were following the dietary patterns (time of intake and amount and macronutrient amount) that were replicated in both nap and no‐nap conditions. Before experimental sessions, participants were asked to refrain from any vigorous physical activity for 24 h prior, during which time they also avoided any alcoholic or caffeine‐containing drinks. Food was consumed on the experimental day at 8:00 and 11: 00 a.m. after the temperature measures were taken (Figure 1). To ensure recovery between trials, there was at least a week between testing conditions for all participants.

### 2.3 Measurements

Prior to the main experimental laboratory sessions, 1RM sessions determined each participant's 1RM percentages for incremental loads of 40, 60, and 80%, allowing a 5‐min recovery between each effort. Two familiarization sessions took place a week after 1RM sessions to ensure the participants were physically capable and the risk of failed efforts during bench press was reduced. The first experimental session took place a week later. Participants underwent two consecutive nights of sleep restriction (02:30 p.m. or 03:30–06:30 p.m.) dependent on the SR group they were allocated to. On the daytime immediately following the second night of SR, participants arrived at the laboratory at 07:30 a.m., 11:00 a.m., 2:00 p.m., and 5:00 p.m. for recordings of intra‐aural temperature (T_IA_) using a thermometer (Genius 1000, Mark 2, Sherwood, Nottingham, UK) and ratings of mood (Profile of Mood State questionnaire; Terry et al. 2003), alertness and tiredness (0–10 cm visual analogue scale) and sleepiness (Karolinska Sleepiness Scale, Akerstedt and Gillberg, 1990). The POMS questionnaire measures mood states with the participants answering 32 questions with eight moods for how they “feel right now.” For the sleep questions, participants ticked on a 10 cm scale their answers “compared to normal,” with ‐5 “being less or earlier,” 0 “normal,” and +5 “more or later.” Additionally, at 5:00 p.m. participants completed the exercise testing. The exercise test session took place at 5:00 p.m. after a standardized active warm‐up. The warm‐up included 5 min on a cycle ergometer (Lode Corival, Furth, Germany) at 150 W followed by a series of dynamic movements that were repeated twice and involved squats (×10), lunges (5 each leg), singleleg Romanian deadlifts (5 each leg), and press‐ups (×10). Immediately post warm‐up, participants had three attempts at grip strength with their right hand, using a dynamometer (Takei Kiki Kogyo, Tokyo, Japan); the highest reading was kept for subsequent analyses. To prepare for the bench press, the force velocity linear encoder (Muscle Lab, Ergotest version 4010, Norway) was attached to a 20 kg Olympic bar to measure displacement (D), average power (AP), peak power (PP), average velocity (AV), and time‐to‐peak velocity (tPV) via a laptop. Five minutes after grip strength measurements, participants undertook three repetitions of bench press at 40, 60, and 80% of their 1RM, with 5‐min rest between each repetition. Sub maximal lifts were recorded using the force transducer, each session performed in the same order of muscle magnitude. At the end of each set for 40, 60 and 80 % 1RM, participants gave a value for their rate of perceived exertion on a 6–20 scale (Borg 1982) for the completed lifts. The highest of the three PP outputs, and associated PV, D, and tPV values, were used for analyses for each mass on the bar (see Figure 1). Between and during conditions, participants were free to live a “normal life,” such as attending lectures, etc. Subject to any protocol restrictions already described (diet, exercise, etc.).

### 2.4 Statistical Analysis

The Statistical Package for the Social Sciences (SPSS IBM) version 29 for Windows was used. All data were checked for normality using the Shapiro–Wilk test. Differences between conditions were evaluated using a general linear model with repeated measures on two factors (nap/no nap condition with two levels, N_0_ and N_60_; “load on bar” with three levels, 40, 60, and 80%) and between‐subject factor (sleep restriction with two levels, SR_3_ and SR_4_). Interaction effects were analyzed between all three variables. To correct violations of sphericity, the degrees of freedom were corrected in a normal way, using Huynh‐Feldt (*ε* > 0.75) or Greenhouse‐Geyser (*ε* < 0.75) values for ε, as appropriate. Graphical comparisons between means and Bonferroni pairwise comparisons were made where main effects were present. The *α* level of statistical significance was set at *p* < 0.05. Effect sizes (d) were calculated from the ratio of the mean difference to the pooled standard deviation (Cohen's *d*). The magnitude of the d was classified as trivial (≤ 0.2), small (> 0.2–0.6), moderate (> 0.6–1.2), large (> 1.2–2.0), and very large (> 2.0) based on guidelines from Batterham & Hopkins (2006). The results are presented as the mean ± the standard deviation throughout the text unless otherwise stated. Ninety‐five percent confidence intervals (95% CI) are presented where appropriate, as well as the mean difference between pairwise comparisons. AU represents arbitrary units. All data is presented as means ± standard deviation or 95% CI for the means in texts, tables, and figures. Significant difference was reflected with *p* ≤ 0.05 and a trend with 0.10 ≤ *p* ≥ 0.05.

## 3 Results

### 3.1 Evening Performance Measures

**FIGURE 2:**
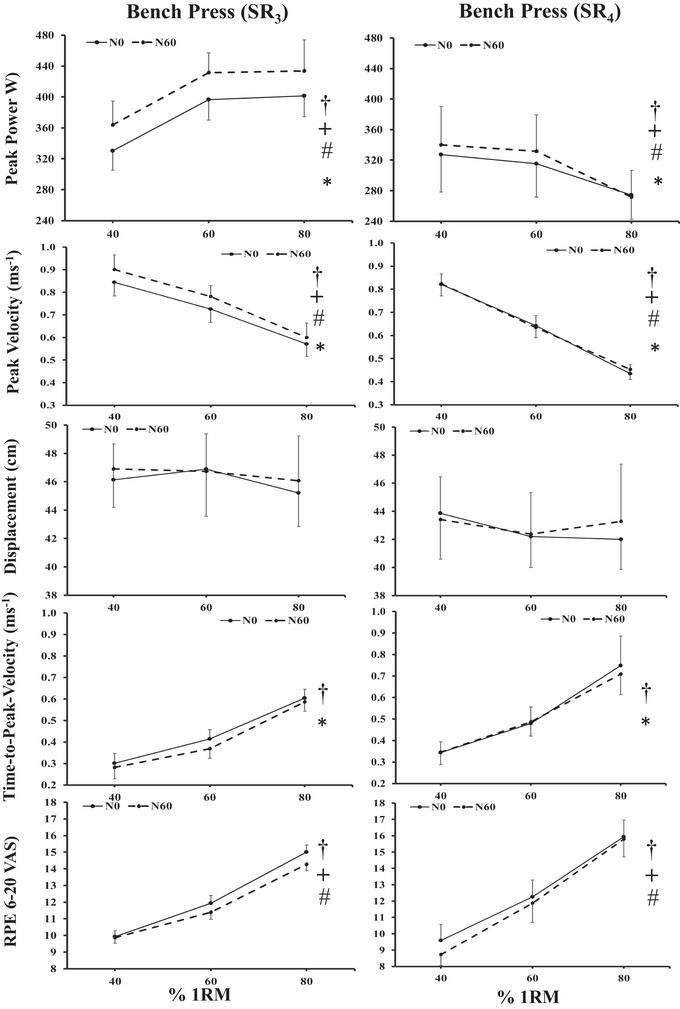
Mean (95% CI) values of each performance variable for evening (5:00 p.m.) bench press at 40, 60, and 80% 1RM loads for the independent groups, either SR_3_ or SR_4_ with two nap conditions (N_0_ and N_60_). † Denotes main effect for load, * main effect for SR, # main effect for N, and + denotes interactions as shown by Bonferroni pairwise comparisons (*p* < 0.05).

**FIGURE 3:**
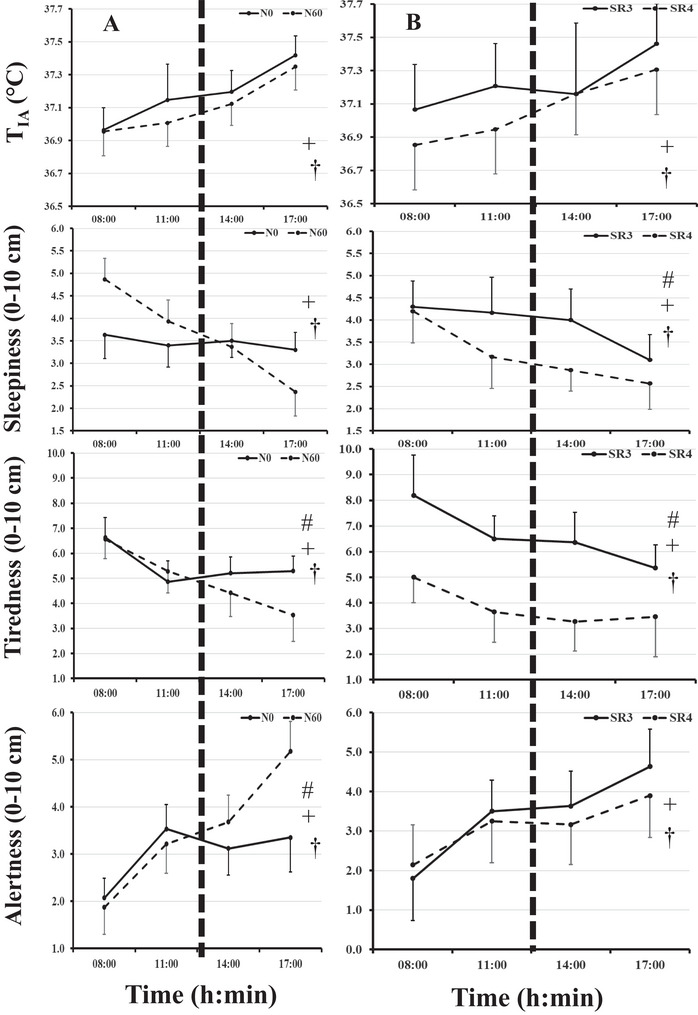
Mean (95% CI) values for intra‐aural temperature, subjective alertness, tiredness, and sleepiness recorded at 08:00 a.m., 11:00 a.m., 2:00 p.m. and 5:00 p.m. for **(i)** *Labeled A*: the Nap conditions (N_0_ and N_60_) and **(ii)** *Labeled B*: the SR conditions (SR_3_ and SR_4_). † Denotes main effect for time‐of‐day, # main effect (N or SR), and + denotes interaction as shown by Bonferroni pairwise comparisons (*p* < 0.05).

Mean ± SD values and the results from the ANOVA statistical analysis are displayed in Tables 2 and 3. Statistical significance of the results can be seen in Figures 2 and 3.

**TABLE 2:** Main effects for sleep restriction (3/4 h SR), nap/no nap (N) conditions, time‐of‐day (TOD) or bar load (LOAD), and interaction effects for intra‐aural temperature and all performance variables measured. *d* = Cohen's *d* effect size and OP = observed power. Bold indicates significant effect (*p* < 0.05); italic indicates a trend (0.05 < *p* < 0.10). AU = arbitrary units. SR_3_, two consecutive nights of sleep restricted to 3h; SR_4_, two consecutive nights of sleep restricted to 4h; N_0_, No nap; N_60_, 60‐min nap at 1:00 p.m.

Variable	Main effect for SR (3 vs. 4)		Main effect for N (0 vs. 60)		Main effect for LOAD/TOD		Interactions (SR * N * LOAD/TOD)
**Intra‐aural temperature (°C)**	*p* = 0.217	*d* = 0.511, OP=23.0%	*p* = 0.122,	*d* = 0.061, OP=33.7%	***p* < 0.001**	***d* = 0.188, OP=100%**	**P = 0.006** TOD*SR
**Right grip Strength (N)**	*p* = 0.438	*d*=0.002, OP=11.8%	*p = 0.062*	*d*=0.310, OP=46.7%			
**Bench Press**							
**Peak‐Power (W)**	***p* = 0.001**	***d*=1.083, OP=93.7%**	***p* = 0.002**	***d*=0.207, OP=91.9%**	***p* = 0.020**	***d*=0.056, OP=67.6%**	p = 0.052 sr*n p < 0.001 load*sr
**Peak‐Velocity (ms^‐1^)**	**p = 0.003**	**d=0.543, OP=88.7%**	**p = 0.009**	**d=0.152, OP=77.5%**	**p < 0.001**	**d=2.86, OP=100.0%**	p = 0.029 sr*n p = 0.013 load*sr
**Displacement (cm)**	*p = 0.070*	d=0.616, OP=44.3%	p = 0.554	d=0.074, OP=89.0%	p = 0.181	d=0.157, OP=5.1%	NA
**Time‐to‐Peak‐Velocity (s)**	**p = 0.009**	**d=0.489, OP=78.0%**	p = 0.411	d=101, OP=12.7%	**p < 0.001**	d=2.515, OP=100.0%	NA

**TABLE 3:** Mean ± SD values for SR_3_ and SR_4_, with *p* values for main effects for SR, N, TOD, and interactions of all tiredness, alertness, sleepiness, and Profile of Mood States (POMS). *d* = Cohen's *d* effect size and OP = observed power. Bold values indicate significant figures; italics indicates a trend (0.1 < *p* > 0.05).

Variables	SR_3_	SR_4_	Significance SR		Significance N		Significance TOD		Significance Interaction
**Tiredness (0–10 VAS)**	6.6 ± 1.7	3.8 ± 2.3	***p* < 0.001**	***d*=0.554, OP=100%**	***p* = 0.009**	***d*=0.203, OP=77.3%**	***p* < 0.001**	**d=0.539, OP=100%**	p = 0.029 n*sr p < 0.001 sr*tod p = 0.016 n*tod p = 0.009 n*tod*sr
**Alertness (0 – 10 VAS)**	3.4 ± 1.7	3.1 ± 2.0	*p* = 0.516	*d*=0.015, OP=9.7%	***p* = 0.027**	***d*=0.162, OP=61.4%**	***p* < 0.001**	**d=0.586, OP=100%**	p = 0.010 n*sr p = 0.089 sr*tod p < 0.001 n*tod p < 0.001 n*tod*sr
**Sleepiness (0 – 10 VAS)**	3.9 ± 1.4	3.2 ± 1.4	***p* = 0.027**	***d*=0.163, OP=61.7%**	*p* = 0.148	*d*=0.073, OP=30.1%	***p* < 0.001**	**d=0.441, OP=100%**	p = 0.066 n* sr p = 0.026 sr*tod p < 0.001 n*tod p < 0.001 n*tod*sr
**Mood State‐Vigor**	5.1 ± 3.0	5.3 ± 3.9	*p* = 0.806	*d*=0.171, OP=5.7%	*p < 0.072*	*d=0.062, OP=43.9%*	**p < 0.001**	**d=1.316, OP=100%**	p = 0.012 sr*tod p < 0.001 n*tod p < 0.001 n*tod*sr
**Mood State‐ Anger**	1.1 ± 1.7	1.3 ± 2.7	*p* = 0.794	*d*=0.363, OP=61.7%	***p* = 0.042**	***d*=0.060, OP=54.1%**	***p* < 0.001**	**d=0.678, OP=95.0%**	NA
**Mood State‐ Tension**	0.4 ± 0.8	0.8 ± 1.6	*p* = 0.212)	*d*=0.177, OP=23.5%	***p* = 0.139**	***d*= 0.310, OP = 31.2%**	***p* < 0.027**	**d=0.496, OP=64.3%**	p < 0.090 n*tod*sr
**Mood State‐ Calm**	6.0 ± 2.9	6.0 ± 3.7	*p* = 0.291	d=0.112, OP=18.0%	*p* = 0.133),	*d*=0.103, OP = 32.1%	***p* < 0.001**	**d=0.634, OP=100%**	p = 0.052 n*sr p < 0.001 tod*sr p = 0.006 n*tod p < 0.001 n*tod* sr
**Mood State‐ Happiness**	5.9 ± 3.0	4.8 ± 3.9	*p* = 0.313	*d*=0.200, OP=16.8%	*p < 0.060*	*d=0.301, OP=47.3%*	***p* < 0.001**	**d=0.800, OP=100%**	p = 0.007 sr*tod p = 0.002 n*tod p = 0.003 n*tod*sr
**Mood State‐ Confusion**	3.3 ± 3.6	1.3 ± 2.2	***p* = 0.037**	***d*=0.232, OP=56.1%**	***p* = 0.002**	**d=0.690, OP=92.2%**	***p* < 0.001**	**d=0.946, OP=100%**	NA
**Mood State‐ Depression**	1.7 ± 2.1	1.2 ± 2.1	*p* = 0.438	*d*=0.412, OP=11.8%	***p* = 0.004**	***d*=0.216, OP=85.2%**	***p* < 0.001**	**d=0.860, OP=99.5%**	NA
**Mood State‐ Fatigue**	10.3 ± 4.7	6.5 ± 4.1	***p* = 0.002**	***d*=0.416, OP=89.7%**	***p* < 0.001**	***d*=0.871, OP=97.8%**	***p* < 0.001**	**d=0.820, OP=100%**	p = 0.014 n*sr p = 0.003 n*tod p < 0.001 n*tod*sr

### 3.2 Grip Strength (right hand)

There was no significant main effect for the SR condition (*p* = 0.438), nor was there any significant main effect for the N condition (*p* = 0.062), nor was there any interaction effect between SR and N for grip strength (*p* = 0.230, Table 2).

### 3.3 Bench Press

A significant effect of the SR condition, with SR_3_ values being higher than SR_4_ values, was observed for PP (+82.9 W, *p* < 0.001, 95% CI: 35.9–129.8 W) and PV (+ 0.102 ms^−1^, *p* = 0.003, 95% CI: 0.039–0.166 ms^−1^). Values of tPV were significantly slower in SR_4_ compared to SR_3_ values (‐0.092 s, *p* = 0.009, 95% CI: 0.026–0.159 s). D and RPE values did not differ between SR conditions (*p* > 0.05). There was a significant main effect of N condition where PP and PV values were all higher (+21.3 W, *p* = 0.002, 95% CI: 8.8–33.8 W; 0.026 ms^−1^ and *p* = 0.009, 95% CI: 0.007–0.045 ms^−1^) and RPE values were all lower in the N_60_ than the N_0_ condition (+0.46 AU, *p* = 0.037, 95% CI: 0.030–0.882 AU). There was a significant interaction between SR^×^N conditions for PV, where values increased with the nap (N_60_) in the SR_3_ condition (+0.05 ms^−1^, *p* = 0.001, 95% CI = 0.020–0.074 ms^−1^) while there was no difference with nap in the SR_4_ condition (*p* > 0.05). There was a significant main effect of “load” for all bench press variables measured (Table 2). PV values were highest at 40% 1RM and lowest at 80% 1RM (0.33 ms^−1^, 0.29–0.38 ms^−1^, *p* < 0.001). PP values were highest at 60 % 1RM and lowest at 40% 1RM (28.5 W, 12.8–44.2 W, *p* < 0.001). Whereas tPV and RPE values were significantly lower at 40% 1RM and highest at 80% 1RM (0.34 s, 0.28–0.41 s, *p* < 0.001; 5.7 AU, 5.2–6.3 AU, *p* < 0.001). Displacement showed no main effect for load (*p* = 0.181). There was a significant interaction of SR^×^ Bar‐load where profiles for PP values for SR_3_ increased from 40 to 80% 1RM, whereas profiles for SR_4_ decreased from 40 to 80%. RPE values increased from 40% to 60% 1RM for both SR_3_ and SR_4,_ with higher values at 80 % load for SR_4_ than SR_3_. PV values were similar at 40% for both SR conditions, but the rate of fall of profiles was greater in SR_4_ than SR_3_ at 80% 1RM (0.38 vs. 0.29 ms^−1^). There was no significant interaction between load^×^ N or between SR^×^ load^×^ N for any variable, such that the values across all conditions for the three loads rose or fell in the same manner (Figure 2, Table 2).

### 3.4 Physiological and Psychological Variables (measured at 08:00 a.m., 11:00 a.m., 2:00 p.m. and 5:00 p.m.)

#### 3.4.1 Intra‐aural Temperature

There was no significant effect of the SR condition, nor a main effect of N on T_IA_ values (*p* < 0.05, Table 2 and Figure 3). There was a significant main effect for time‐of‐day (*p* < 0.001) on T_IA,_ where values increased from 08:00 a.m. to 5;00 p.m. (mean difference = 0.42°C; *p* < 0.001; 95% CI = 0.35–0.50°C). There was an interaction for SR^×^ time‐of‐day where T_IA_ values increased from 08:00 a.m. to 5:00 p.m. for the SR_4_ group, while the SR_3_ profile increased from 08:00 to 11:00 a.m. and then flattened after the nap at 2 p.m. to increase at 5:00 p.m. There were no other significant interactions (*p* > 0.05, Table 2; Figure 3).

#### 3.4.2 Tiredness and Alertness

There was a significant effect for SR on subjective tiredness, with higher scores respectively for SR_3_ versus SR_4_ (+2.6 cm, p < 0.001, 95% CI = 1.7–3.6 cm), but not alertness (*p* = 0.516, Table 3). A significant main effect for N was found, where ratings of tiredness were higher and alertness values lower without the nap (+0.55 cm, 95% CI = 0.15–0.97 cm, *p* = 0.009 and ‐0.47 cm, 95% CI = 0.06‐0.88 cm, *p* = 0.027, respectively). There was a significant interaction for SR^×^N where ratings of tiredness and alertness with the nap (N_60_) in the SR_3_ improved values greater than SR_4_ (1.0 cm, 95% CI = 0.44–1.59 cm; *p* = 0.001 and 1.0 cm, 95% CI = 0.44–1.60 cm; *p* = 0.001). No difference between the SR_3_ and SR_4_ was observed for ratings of tiredness in the N_0_ condition (*p* = 0.725 and *p* = 0.771). There was a significant time‐of‐day effect for ratings of tiredness and alertness, where tiredness values where highest in the morning and lowest in the evening respectively, with the opposite profile for alertness (2.7 cm, 95% CI = 1.50‐3.80 cm; *p* = 0.001 and 2.3 cm, 95% CI = 1.84–2.76 cm; *p* < 0.001). There was a significant interaction for time‐of‐day*SR for tiredness and alertness, where profiles of tiredness for SR_3_ fall from 08:00 a.m. to 5:00 p.m. (2.8 cm, 95% CI = 2.05–3.62 cm; *p* = 0.001_Figure 3), while the profile for SR_4_ falls from 08:00 a.m. to 2:00 p.m. (2.8 cm, 95% CI = 2.05‐3.62 cm; *p* = 0.001; Figure 4), then stayed steady from 2:00 to 5:00 p.m. with values being highest in the morning and lowest in the evening (2.7 cm, 95% CI = 1.50–3.80 cm; *p* = 0.001). Profiles for alertness increase similarly from 08:00 a.m. to 2:00 p.m. with greater alertness at 5:00 p.m. in the SR_3_ than SR_4_ condition (Figure 4). There was a significant interaction for time‐of‐day^×^Nap where profiles of tiredness decreased in N_0_ or increased for alertness values from 2:00 to 5:00 p.m. (0.87 cm, *p* = 0.001, 95% CI = 0.38–1.35 cm and 1.50 cm, 95% CI = 0.86–2.14 cm, *p* < 0.001, Figure 3) and stayed the same for N_60_ for both tiredness and alertness (*p* > 0.05). There was a significant interaction for time‐of‐day^×^N^×^SR for tiredness and alertness values (*p* < 0.05), where alertness and tiredness profiles for both variables improved (increased and decreased, respectively) after the nap (2:00–5:00 p.m.) in the SR_3_ condition only.

#### 3.4.3 Profile of Mood State

There was a significant effect of SR for mood state values, where confusion and fatigue values were higher in the SR_3_ than the SR_4_ condition (1.95 AU, *p* = 0.025, 95% CI = 0.27–3.64 AU and 3.84 AU, *p* = 0.002, 95% CI = 1.49–6.20 AU, respectively). There was a significant effect of N where scores of fatigue, depression, confusion, and anger were higher in the N_0_ than N_60_ condition (1.96 AU, *p* < 0.001, 95% CI = 0.99–2.93 AU; 0.83 AU, *p* = 0.004, 95% CI = 0.29–1.38 AU; 0.72 AU, *p* = 0.002, 95% CI = 0.29–1.14 AU; 0.78 AU, *p* = 0.042, 95% CI = 0.03–1.54 AU). There was a significant interaction for N*SR where, for the SR_3_ condition, the nap reduced fatigue scores and increased vigor, happiness, calmness, and vigor scores (Table 3, *p* < 0.05). There was a significant effect of time‐of‐day for vigor, anger, tension, calm, happiness, confusion, depression, and fatigue (*p* < 0.05; see Table 3). Anger, tension, confusion, and depression were significantly higher in the morning compared to the evening, whereas vigor, calm, and happiness were significantly higher in the morning than in the evening (see Table 3). There was a significant interaction for time‐of‐day^×^SR where SR_3_ profile demonstrated increased ratings for vigor, calmness, and happiness from 08:00 a.m. to 5:00 p.m. and decreased rating for fatigue between 08:00 a.m. and 5:00 p.m., while in SR_4_ scores for vigor, calmness, happiness, and fatigue were similar from 08:00 a.m.to 5:00 p.m.17:00 h. There was a significant interaction for time‐of‐day×N, where all variables show similar profiles from 08:00 a.m.to 5:00 p.m.; thereafter, for the nap condition values (N_0_) there is a corresponding increase in vigor, happiness, and calmness and a decrease in fatigue values compared to the no nap condition (N_60_; Table 3). There was a significant interaction for time‐of‐day^×^N^×^SR with N_60_ scores for vigor, calmness, and happiness increasing and ratings of fatigue decreasing compared to no nap (N_0_) in the SR3 condition only.

#### 3.4.4 Stanford Sleepiness Questions

There was a significant effect of SR for sleepiness values, where subjective responses were higher in the SR_3_ than SR_4_ condition (0.69, *p* = 0.027, 95% CI = 0.09–1.30 AU—see Table 3 and Figure 3). There was no main effect of N for sleepiness nor interaction of N*SR (*p* > 0.05). There was a significant effect of time‐of‐day for sleepiness, where values were higher in the morning and decreased in the evening (1.42 AU, *p* < 0.001, 95% CI = 1.10–1.73). The time‐of‐day mood profiles were evident from 08:00 a.m. to 5:00 p.m. There was a significant interaction for time‐of‐day^×^SR length, where profiles for SR_3_ values are similar from 08:00 a.m. to 2:00 p.m. and then drop to 5:00 p.m., but for SR_4_ values drop from 08:00 a.m. to 5:00 p.m. (*p* = 0.026). There was a significant interaction for time‐of‐day^×^N where profiles of sleepiness dropped from 08:00 a.m. to 5:00 p.m. in the N_60_ condition (with nap) but stayed the same from 2:00 to 5:00 p.m. in the N_0_ condition. There was a significant interaction for time‐of‐day^×^N^×^SR where profiles of sleepiness were higher from 08:00 to 11:00 a.m. in the SR_3_ than the SR_4_ condition, but after the nap (at 1:00 p.m.) sleepiness fell to the lowest values at 5:00 p.m. In N_0_ condition, levels of sleepiness were higher in SR_3_ than SR_4_ from 2:00 to 5:00 p.m.

## 4 Discussion

We report a significant interaction with increased peak‐velocity (+ 6.2%, *p* = 0.029) and a trend for peak‐power values (+ 8.2 %, *p* = 0.052) for bench press at 17:00 h, after having had a 60‐min nap (N_60_) in the SR_3_ (two‐consecutive nights of 3‐h sleep; Figure 2) condition only. This was accompanied with corresponding improvements in mood‐states (happiness, calmness and fatigue) and subjective states (tiredness, alertness, and sleepiness) in the SR_3_ condition after the nap (2:00 p.m.–5:00 p.m.) to values like that of the SR_4_ condition. Further, regardless of taking a nap or not, the profiles of bench press variables and RPE for the three loads, were similar in the SR_4_ condition (Figure 2). However, to maintain independent analysis, we did not include a control condition (3 h of habitual sleep taken for two nights) in the present protocol. From the first analysis of the data, it was reported that a 1‐h post‐lunch nap was effective in the 3‐h SR condition in restoring strength and subjective ratings compared to normally taken sleep values at 5:00 p.m. (Brotherton et al., 2019). It is also unclear whether the 4‐h SR condition presented enough stress‐strain adaptation to reduce submaximal bench press values, compared to habitual sleep. Such methodological limits have previously been suggested to partially explain the lack of effect between having a nap compared to no nap on muscle strength parameters (Gallagher et al., 2023). Further, subjective levels of alertness, sleepiness, and levels of mood state for confusion and fatigue were positively affected by the 4‐h versus 3‐h SR (*d* = 0.02 to 0.55, trivial to moderate effect sizes; Table 3). With higher values for alertness, and lower for sleepiness, confusion, and fatigue, respectively. These unique findings suggest a dose‐response for sleep‐loss for these variables, with 37.3% for two nights negatively affecting strength as well as subjective measures compared to a 49.2% loss of habitual sleep (assessed from the participants normally reported sleep of ∼8.1 decimal hours per night; Table 1). Where the intervention of a 60‐min nap restored strength and subjective mood, etc. to similar levels as that of a 50% sleep loss. Like Belenky et al. (2003), we used independent groups for sleep loss conditions, rather than employing a research design where one group of participants completes all four conditions. A transient fall in core body temperature and alertness is expected around 11:00 a.m.–2:00 p.m. (post‐lunch dip) when partially sleep deprived (Waterhouse et al. 2007). However, we found this post‐lunch dip was only evident for the greater sleep loss condition and not affected by a nap, again supporting a dose‐response to sleep loss that is reflected by core temperature changes (Figure 3).

A dose‐response to sleep loss effects has been shown to negatively affect mood, a psychomotor vigilance test or cognitive performance (in the domain of attention, working memory, and executive function), as well as metabolic function and psychological health (Van Dongen et al., 2003; Belenky et al., 2003; Banks et al., 2010; Lim & Dinges, 2010; Goel et al., 2009). Like the findings of the current investigation, Belenky et al. (2003) reported a threshold of < 4h sleep duration per night resulted in decrements in levels of alertness and psychomotor vigilance performance. However, unlike the current study, the habitual sleep of participants by Belenky et al. (2003) was not reported; hence, participants could have regularly had restricted sleep and would not be as sensitive to those who achieve recommended hours of sleep. Further comparisons between our and others work are difficult, as there is no research on a dose effect of sleep‐loss of habitual sleep on muscular or weightlifting performance, considering greater sleep loss leads to bigger decreases in performance (Silva et al., 2021; Walsh et al., 2021; for recent reviews). We have previously stated there may be a cut off wherein in our case 50% of habitual sleep taken for two nights is tolerated, and the homeostatic drive is not affected by a 30‐ or 60‐min nap compared to no nap (Gallagher et al., 2023). Hence, we hypothesized that when partially sleep restricted to 3h, it would decrease strength performance measures compared to the 4‐h condition, irrespective of nap/no nap condition. However, we found that bench press PP and PV were generally higher in the SR_3_ versus SR_4_ condition and time‐to‐peak‐velocity lower (took less time), respectively. Interactions of “load and SR” were present for PV and PP, where PV reduction with increasing load was more pronounced in the SR_4_ than the SR_3_ group. Further, PP values increased from 40–80% 1RM in the SR_3_ group compared to the SR_4_ group, whose profile showed a reduction from 40‐80 %. Although these populations were similar in terms of strength conditioning (> 2 vs. > 2 years), sleep habits (∼8.1 vs. ∼8.2‐h), age (22.7 ± 2.5 vs. 21.6 ± 1.6 years), chronotype (all intermediate types), and other individual differences such as all flexibility and vigorous questionnaire scores (all flexible and rigid) the 1RM values for the participants of the SR_3_ condition were higher than that of the SR_4_ group (21.5% or 17.3 kg). This difference in 1RM values may have contributed to the observed differences in submaximal strength indices profiles with a shifting of the load‐velocity and load‐power profile, with the optimal load for power output varying depending on individual factors (training status, strength level, and experience). Although we counterbalanced the participants for the order of nap/no nap conditions as suggested to reduce bias, we did not match the participants for a strength variable and then allocate them to groups based on the minimized method that would have removed this discrepancy of 1 RM (Edwards et al., 2024c).

Our second aim was to investigate the effectiveness of a 60‐min post‐lunch nap (N_60_), compared to no nap (N_0_), and whether it would improve evening physiological and subjective psychological measures. We found improvements following the nap regarding ratings of alertness, sleepiness, and mood states such as confusion and fatigue. With corresponding increases in peak‐power (+ 21.3 W), peak velocity (+ 0.026 ms^−1^) and ratings of perceived exertion (+ 0.46 AU; Table 2, Figure 2). These findings agreed with our hypothesis that a nap taken after repeated partial sleep loss would improve performance measures. As no “control session” has been included in the present protocol, it is difficult to conclude that a nap would be an easy strategy to restore the ability to perform. However, as the positive effect of the 60‐min nap was observed on PP, PV and tPV only in the group with larger sleep restriction (i.e., larger homeostatic imbalance), it could be hypothesized that as sleep deprivation increases, so does the accumulation of endogenous hypnogenic substances in the brain (Borbély and Tobler, 1989), which could be eliminated at least in part during the nap. Therefore, the beneficial effect of a nap on performance indices would be “more visible” in the group with larger sleep restriction. The lack of change as grip strength with sleep restriction agrees with others where maximum strength appears not to be affected by loss in habitual sleep; hence, a nap should have no effect (Reilly and Edwards 2007; Edwards et al., 2023). Higher “skill‐orientated” submaximal lifts with a high cognitive component (such as bench press) are thought to be affected more by sleep loss compared to less skill‐oriented maximal tasks such as grip strength (Gallagher et al., 2023; Edwards et al., 2023). This reduction in cognitive tasks after 1:00 p.m. has been attributed to higher catecholamine levels in the blood, hence increased arousal and a corresponding increase in homeostatic drive due to time‐awake causing mental fatigue (Reilly and Edwards 2007; Carrier and Monk 2000).

### 4.1 Limitations

The absence of a control sleep condition (8h) and other levels of sleep loss, such as 2h, meant that the full dose‐response on sporting performance (bench press) could not be evaluated for a critical threshold. This is a secondary analysis of two previously published studies, and rather than complicate the analysis, no control conditions of normal sleep were included. Our working group has previously reported effects of naps with no sleep loss/restriction on similar variables (Brotherton et al., 2019). Last, no measures to assess “sleep” immediately before the start of the experiment and during the two nights were taken, such as actigraphy monitoring of sleep/wake patterns. Nor could the proposed mechanisms that enable sleep be taken during the “nap” period or the “restricted sleep,” and therefore we relied on subjectively reported quality. Polysomnography, temperature changes between the core and skin (heat loss/gain), and changes in secretory melatonin would have allowed further understanding of the effects of restricted sleep, mechanisms, and effectiveness of the nap. Especially in respect to total sleep duration, sleep latency, and sleep efficiency which are reported to be markers of sleep quality when efficiency is greater than 85% (of the time‐in‐bed) and latency less than 15 min (Ohayon et al., 2017). We acknowledge that although we controlled the exposure to daylight prior to entering the laboratory, by conducting the experiment in winter in the United Kingdom, we did not provide sufficient control over light conditions during the experiment, which could impact the outcome measures, particularly the subjective ratings. However, as the participants were all students at the university and the experimental day was the same for each week, the participants would have had to attend the same lectures and hence have similar structure to their days (including indoor or outdoor light exposure). Last, in this study we recruited only male participants, we acknowledge this is a limitation of the study. Healthy females have been shown to respond to sleep loss differently than males, in terms of an advance in their tiredness rhythm (Edwards, 2025). We acknowledge that this area of this research area is interesting, as such gender differences may have implications on the submaximal muscle performance (such as bench press variables). Where females are hypothetically affected cognitively and physically more in the evening than males, and this may be worsened by greater amounts of sleep loss.

## 5 Conclusion

A dose‐response relationship between sleep loss and submaximal bench press performance was found, where PP, PV, and tPV were negatively affected (decreased or increased, respectively) with SR_4_ versus SR_3_. This was not in the expected direction but was rather due to greater general strength in the SR_3_ group but was accompanied by lower values of tiredness, fatigue, confusion and sleepiness and an increase in alertness in the SR_4_ group than SR_3,_ reflecting the negative effects of greater sleep loss on tiredness, alertness, sleepiness, and mood. Time‐of‐day effects were shown in intra‐aural temperature, subjective, and mood responses (*p* < 0.05). Positive modifications in sleepiness, alertness, and tiredness after the 1‐h nap at 1:00 p.m. were found. With a corresponding improvement in PV and a trend in PP (*p* = 0.029 and *p* = 0.052) after the 1‐h nap, but only in the SR_3_ condition.

## Author Contributions

**Ben Joseph Edwards**: conceptualization, investigation, funding acquisition, writing – original draft, writing – review and editing, visualization, validation, methodology, software, formal analysis, project administration, resources, supervision, and data curation. **Ellis Brotherton**: conceptualization, investigation, writing – original draft, writing – review and editing, methodology, formal analysis, project administration, supervision, and data curation. **Adrian Markov**: conceptualization, investigation, writing – original draft, methodology, validation, writing – review and editing, formal analysis, data curation, and supervision. **Theresa Toussaint**: supervision, formal analysis, methodology, visualization, writing – review and editing, writing – original draft, and investigation. **Magali Giacomoni**: investigation, writing – original draft, writing – review and editing, validation, methodology, formal analysis, data curation, and project administration. **Chloe Gallagher**: conceptualization, investigation, writing – original draft, writing – review and editing, validation, software, formal analysis, resources, supervision, and data curation. **Samuel Andrew Pullinger**: conceptualization, investigation, writing – original draft, writing – review and editing, validation, methodology, formal analysis, supervision, and data curation.

## Author Contributions

Ellis Brotherton: Conceptualization; data curation; formal analysis; funding acquisition; investigation; methodology; validation; visualization; writing ‐ original draft; writing ‐ review and editing. Adrian Markov: Data curation; formal analysis; validation; visualization; writing ‐ original draft; writing ‐ review and editing. Theresa Toussaint: Data curation; formal analysis; validation; visualization; writing ‐ original draft; writing ‐ review and editing. Magali Giacomoni: Data curation; formal analysis; funding acquisition; methodology; supervision; validation; visualization; writing ‐ original draft; writing ‐ review and editing. Chloe Gallagher: Data curation; formal analysis; funding acquisition; investigation; methodology; project administration; resources; supervision; validation; visualization; writing ‐ original draft; writing ‐ review and editing.

## Conflicts of Interest

The authors report no conflicts of interest, the linear encoder was bought from internal funds and there is no link between our laboratory and chronobiology group and the MuscleLab Company. The authors are responsible for the content and writing of this article.

## Peer Review

The peer review history for this article is available at https://publons.com/publon/10.1002/brb3.70741

## Data Availability

Data is unavailable due to ethical restrictions.
